# Predictors of subclinical diastolic dysfunction measured by MRI: multi-ethnic study of atherosclerosis (MESA)

**DOI:** 10.1186/1532-429X-11-S1-O15

**Published:** 2009-01-28

**Authors:** Sadia Qadir, Wendy S Post, Gregory W Hundley, Gregory DN Pearson, Shantanu Sinha, Joao Lima, David A Bluemke

**Affiliations:** 1 Massachusetts General Hospital, Boston, MA USA; 2 Johns Hopkins Hospital, Baltimore, MD USA; 3 Wake Forest University School of Medicine, Winston-Salem, NC USA; 4 Columbia University, New York, NY USA; 5 UCLA School of Medicine, Los Angeles, CA USA; 6 National Institute of Health, Bethesda, MD USA

**Keywords:** Diastolic Dysfunction, Retrospective Study Design, Peak Filling Rate, Diastolic Hypertension, Exclusive Reliance

## Introduction

Diastolic dysfunction, often preclinical with no recognized CHF diagnosis, is associated with marked increases in all-cause mortality. Current data on diastolic dysfunction have limitations due to retrospective study designs and/or exclusive reliance on echocardiography.

## Purpose

The purpose of this study was to analyze the predictors of diastolic dysfunction in the MESA population using cardiac MRI.

## Methods

We studied peak filling rate (PFR ml/s) and time to peak filling (TPFR msec) in a subclinical population (n = 4465, males 47%, mean age 62 ± 10 years). Mean (SD), correlation coefficients and multivariable regression coefficients were determined.

## Results

**Figure 1:**
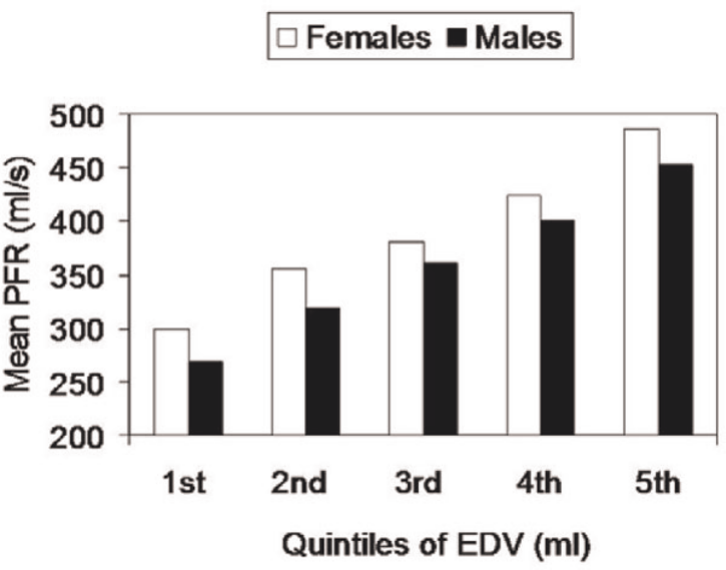
Figure 1

Table 1 illustrates associations between risk factors with diastolic LV function. End diastolic volume (EDV) modified the relationship of gender and PFR. Mean PFR was therefore analyzed across quintiles of EDV and was found to be higher in females 386.35, [95%CI 382.53 to 390.17] compared to males 359.11 ml/s [95%CI 355.05 to 363.17] (Fig 1). Comapred to non-smokers smokers had lower peak filling rates. Compared to Whites, Hispanics were at a higher risk for diastolic dysfunction, Chinese ethnicity showed a relative protective effect after adjusting for all other risk factors.

**Table 1:** Multivariable regressions analysis of predictors of diastolic LV dysfunction

Independent Predictors	Peak Filling Rate (ml/s)			Time to Peak Filling (msec)		
	Regression Coefficient	95% CI	P-Value	Regression Coefficient	95% CI	P-Value
Age (years)	-1.70	-1.20 to -1.40	<0.001	1.94	1.53 to 2.36	<0.001
DBP (mmHg)	-0.42	-0.78 to -0.05	0.02	0.48	0.14 to 0.82	0.005
HTN meds	-5.92	-11.18 to -0.62	0.03	8.86	3.01 to 14.71	0.003
BMI (kg/m^2^)	-0.67	-1.32 to -0.05	0.04	1.37	0.62 to 2.11	<0.001
Former Smokers	-6.52	-12.30 to 0.65	0.03	1.00	-4.56 60 6.58	NS
Impaired Fasting Glucose	2.63	-2.74 to 8.01	NS	9.06	3.21 to 17.38	0.03
Chinese	8.14	-0.25 to 16.53	NS	-8.19	-14.27 to -1.87	0.01
Hispanics	8.33	1.88 to 14.88	0.006	4.67	-1.76 to 11.1	NS

## Conclusion

Impaired LV relaxation is associated with increasing age, male gender, obesity, diastolic hypertension, smoking, and varied by ethnicity.

